# Transfusion of female blood in a rat model is associated with red blood cells entrapment in organs

**DOI:** 10.1371/journal.pone.0288308

**Published:** 2023-11-22

**Authors:** Abdulrahman Alshalani, Marit B. de Wissel, Anita M. Tuip-de Boer, Joris J. T. H. Roelofs, Robin van Bruggen, Jason P. Acker, Nicole P. Juffermans

**Affiliations:** 1 Chair of Medical and Molecular Genetics Research, Department of Clinical Laboratory Sciences, College of Applied Medical Sciences, King Saud University, Riyadh, Saudi Arabia; 2 Laboratory of Experimental Intensive Care and Anesthesiology, Amsterdam UMC, University of Amsterdam, Amsterdam, The Netherlands; 3 Department of Pathology, Amsterdam UMC, University of Amsterdam, Amsterdam, The Netherlands; 4 Amsterdam Cardiovascular Sciences, Microcirculation, Amsterdam, The Netherlands; 5 Department of Molecular Hematology, Sanquin Research and Landsteiner Laboratory, Academic Medical Center, University of Amsterdam, Amsterdam, The Netherlands; 6 Department of Laboratory Medicine and Pathology, University of Alberta, Edmonton, Alberta, Canada; 7 Innovation and Portfolio Management, Canadian Blood Services, Edmonton, Alberta, Canada; 8 Department of Intensive Care, OLVG Hospital, Amsterdam, the Netherlands; Australian Red Cross Lifeblood, AUSTRALIA

## Abstract

Transfusion of red blood cells (RBCs) has been associated with adverse outcomes. Mechanisms may be related to donor sex and biological age of RBC. This study hypothesized that receipt of female blood is associated with decreased post-transfusion recovery (PTR) and a concomitant increased organ entrapment in rats, related to young age of donor RBCs. Donor rats underwent bloodletting to stimulate production of new, young RBCs, followed by Percoll fractionation for further enrichment of young RBCs based on their low density. Control donors did not undergo these procedures. Male rats received either a (biotinylated) standard RBC product or a product enriched for young RBCs, derived from either male or female donors. Controls received saline. Organs and blood samples were harvested after 24 hours. This study found no difference in PTR between groups, although only the group receiving young RBCs from females failed to reach a PTR of 75%. Receipt of both standard RBCs and young RBCs from females was associated with increased entrapment of donor RBCs in the lung, liver, and spleen compared to receiving blood from male donors. Soluble ICAM-1 and markers of hemolysis were higher in recipients of female blood compared to control. In conclusion, transfusing RBCs from female donors, but not from male donors, is associated with trapping of donor RBCs in organs, accompanied by endothelial activation and hemolysis.

## Introduction

Transfusion of red blood cells (RBCs) has been associated with adverse outcomes [1–6]. Observational studies have suggested an association between receipt of female blood, but not of male blood, and mortality [7–9], although not all studies concur [10, 11]. Thereby, donor sex may impact transfusion outcomes, but biological mechanisms are not known.

Blood products contain RBCs with a wide age spectrum, spanning from newly matured reticulocytes which express the transferrin receptor (CD71), to four month-old RBCs [12]. Reticulocytes, and possibly also other young RBCs, retain phenotypic properties which are thought to contribute to immunomodulatory effects [13–15]. The age distribution of RBCs in blood products is dependent on donor sex [16]. Female blood products contain RBCs with higher cell volume and slower cellular aging properties than male donors, indicating that female donors have more young RBCs at the time of donation compared to male donors [17]. In addition, female donor blood yields a lower hemoglobin increment compared to recipients of male RBCs [18], possibly due to a lower hemoglobin level compared male products [19] or due to enhanced clearance of female RBCs from recipient’s circulation [16]. Given that female blood contains more circulating young RBCs, which may be more immunogenic, we hypothesized that transfusing a female RBC product or an RBC product enriched for young cells, would cause a lower PTR after 24 hours of transfusion due to enhanced clearance of transfused RBCs from the circulation of recipients as compared to males, with concomitant entrapment of the cleared RBCs in organs and poor blood quality parameters. We choose to study PTR as it is an important quality measure for RBC products, calculated as the survival of transfused RBCs in the circulation of recipients 24 hours after transfusion [20]. Current standards for human RBC products dictate a PTR of at least 75% [21], which have been used as a quality measurement in animal studies [22, 23].

## Study design and methods

### Study groups

**Fig 1:**
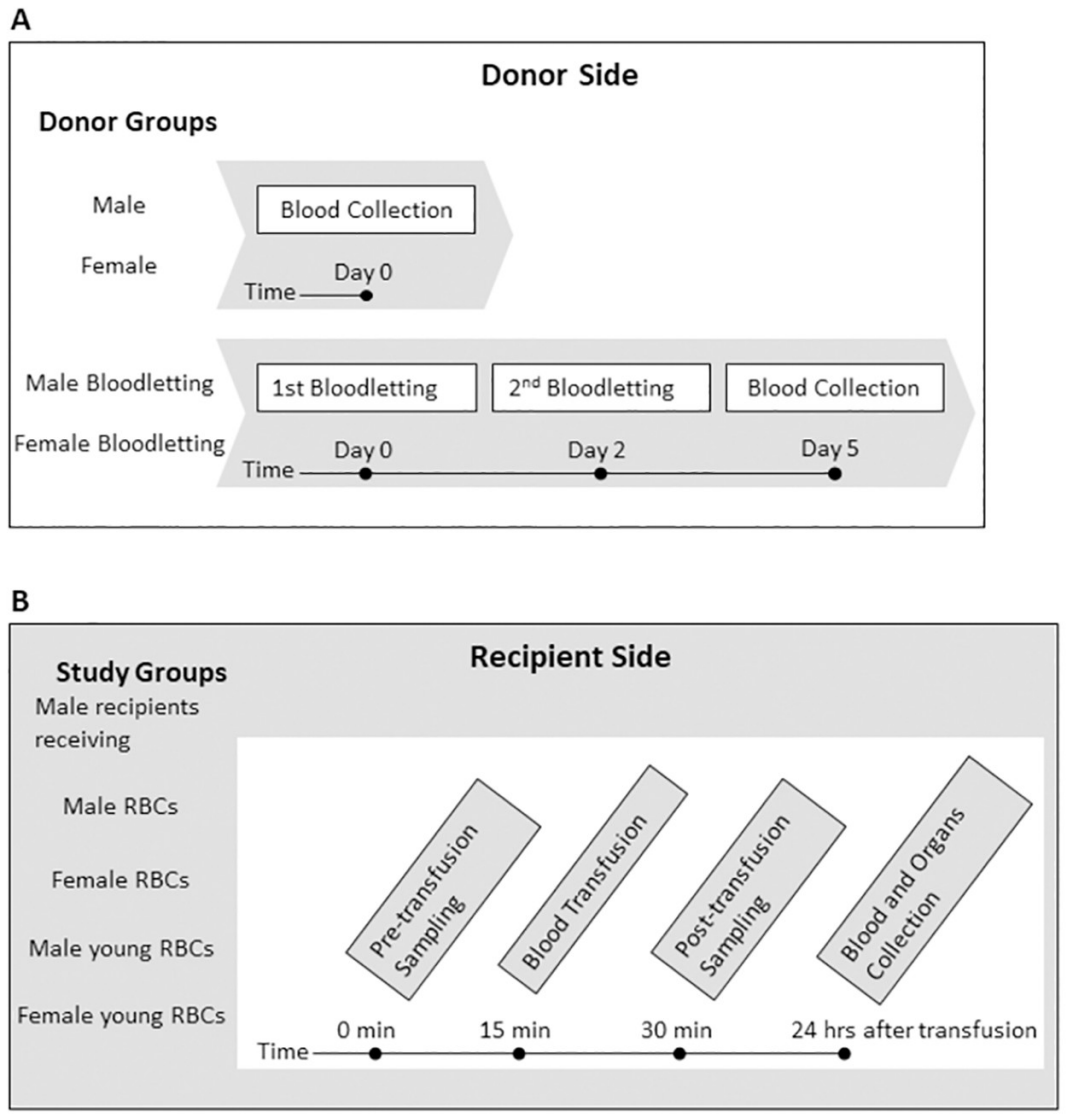
Flowchart of the study design for (A) donor and (B) recipient groups.

This study was approved by the Animal Care and Use Committee of Amsterdam University Medical Center, location AMC, Amsterdam, the Netherlands. Volumes of blood samples and blood transfusions were estimated in accordance with the animal practice guidelines [24]. Adult Sprague Dawley rats of 12 to 13 weeks old were purchased from Envigo laboratory (the Netherlands) and kept in the animal facility until the time of the experiment. Donors were divided into four groups; male, female, male bloodletting, and female bloodletting (Fig 1). All recipients were male rats and divided into five groups; control group received normal saline (0.9% NaCl; B Braun), recipients of male standard RBCs, recipients of female standard RBCs, recipients of male young RBCs, and recipients of female young RBCs (n = 10 for all groups).

### Enriching blood products for young RBCs

Bloodletting was performed to stimulate production of new RBCs and, therefore, increase the ratio of young RBCs in the circulation. Bloodletting was done twice (at day 0 and day 2 for bloodletting donor groups) by removing 10% of the total blood volume from the lateral tail vein (2.1 mL for males and 1.5 mL for females at each bloodletting time point) while under anesthesia with 4% isoflurane (Pharmachemie BV).

At the time of blood collection, donor rats were anesthetized with 4% isoflurane, and animals were bled through transthoracic heart puncture in a syringe containing 1:10 CPD. Blood was centrifuged (1100 xg, 10 min, 20°C) and washed two times with SAGM (Saline-Adenine-Glucose-Mannitol) additive solution. RBCs were then suspended in an equal volume of SAGM, pooled together, and leukoreduced using an Acrodisc WBC syringe filter (Pall Laboratory). To further increase the fraction of young RBCs, a Percoll fractionation method was used as described previously [17], with modifications. Briefly, the appropriate Percoll density was determined for each transfusion product by gently layering 0.75 mL of each RBC product on 2 mL of different Percoll (Cytiva) densities (1.093, 1.095, 1.098, 1.100, 1.103, and 1.103 g/L) in 5 mL tubes (BD Falcon). Tubes were centrifuged (2200 xg, 10 min, 20°C) with low acceleration and deceleration speeds (2 and 1 correspondingly), and the appropriate Percoll density was visually determined for each sample by choosing the lowest density that led to a clear separation of young RBCs and old RBCs. Then, young RBCs were isolated by gently layering 1.5 mL of RBC on 3 mL of the corresponding appropriate Percoll density in 5 mL tubes. Tubes were centrifuged with the same centrifuge settings, and the top layer (young RBCs) was isolated. Cells were washed two times in SAGM to remove residual Percoll and suspended in an equal volume of SAGM. Standard RBCs of non-bloodletting donor groups and young RBCs of bloodletting donor groups were then biotinylated (concentration 48 μg/mL; EZ-Link Sulfo-NHS-LC-Biotin; Thermo Scientific) as described previously [23]. Transfusion products were stored at 4°C until the time of transfusion on the following day. Standard blood products were prepared following heart puncture by the same protocol but without prior blood letting or the percoll separation.

### Experimental protocol

The day following blood product preparation, recipients were anesthetized with 4% isoflurane. The tail vein was cannulated with a Vasofix catheter (B Braun). A pre-transfusion sample (0.5 mL) was collected from the cannula. After 15 min, a transfusion product, or normal saline for the control group, of 2.1 mL per recipient was infused through the cannula. A post-transfusion sample (0.5 mL) was collected from the cannula 15 min later. The catheter was then removed, and rats were allowed to wake up. After 24 h of blood transfusion, rats were anesthetized with 4% isoflurane, and sacrificed by exsanguination via transthoracic heart puncture. Blood samples were taken for PTR. The rest of the blood was centrifuged (1500 xg, 10 min, 20°C), and the supernatant was frozen at -80°C for further plasma analyses. The right lung, the upper two lobes of liver, and the spleen were removed and divided into two parts, from which one part was fixed with 10% formaldehyde and embedded in paraffin for histological assessment. The other part of the organs was collected in 15 mL 1 x PBS, homogenized, and filtered for the entrapment of donor RBCs in organs.

### Blood assays

RBC indices were measured using Coulter Counter (Beckman Coulter), which include RBC count, hematocrit (Htc), hemoglobin level (Hb), mean corpuscular volume (MCV), mean corpuscular hemoglobin (MCH), mean corpuscular hemoglobin concentration (MCHC), red blood cell distribution width (RDW). Blood gas analyses were measured using Rapidpoint 500 blood gas analyzers(Siemens Healthcare Diagnostics) and included pH, partial pressure of oxygen (pO_2)_, partial pressure of carbon dioxide (pCO_2_), lactate concentration, sodium (Na^+^) concentration, and potassium (K^+^) concentration. Reticulocyte percentage was determined by presence of CD71^+^ cells, following incubation of RBCs with a saturating concentration of antibody mixture containing anti-CD71 (FITC; Biolegend) and anti-rat erythroid cells (PE; Biolegend) for 30 min at 4°C in the dark. PTR was estimated from the fraction of biotinylated RBCs that remain in the circulation at 24 h compared to the sample taken directly post transfusion. The entrapment of donor RBCs in organs was determined from the percentage of biotinylated donor RBCs in organs compared to the percentage of biotinylated donor RBCs in the circulation. To determine the percentage of biotinylated RBCs in recipient blood and organ, 1 μL of blood or cells from homogenized organs were incubated with a saturating concentration of antibody mixture containing streptavidin (Alexa Fluor 647; Invitrogen) and anti-rat erythroid cells (PE; Biolegend) for 30 min at 4°C in the dark. Labeled cells for reticulocyte percentage, PTR, and donor RBCs in organs were washed twice, suspended in 200 μL of HEPES buffer, and acquired using the BD FACSCanto Flow Cytometer (BD Bioscience). The analysis was performed with FlowJo v10 software (FlowJo, Ashland, OR).

### Plasma assays

Levels of endothelial markers of intercellular adhesion molecule-1 (ICAM-1) and von Willebrand factor (vWF) in the plasma were measured by enzyme-linked immunosorbent assay (ELISA) kits (ICAM-1 form R&D systems and vWF from Elabscience). Free hemoglobin concentration was determined spectrophotometrically (Lambda 365 UV/Vis, Perkin Elmer) from absorption at wavelengths of 562, 578, and 598 nm, as described previousely [25]. Haptoglobin concentration was measured by an immunoturbidimetric assay (Cobas c702, Roche).

### Histological assessment

Four-micrometer thick paraffin sections of lung, liver and spleen tissue were stained with hematoxylin and eosin, blinded, and analyzed by a pathologist, as described previously [23] Spleen tissue was stained with Perls Prussian blue to assess the iron accumulation in the spleen. The analysis of iron accumulation was performed with QuPath version 0.2.3 [26].

### Statistical analysis

A power analysis was done based on a previous study on the impact of washing of RBCs with PTR as an outcome [23] yielding 10 animals per group with 80% power to detect a 10% difference in PTR with a standard deviation of 15%. The normality of the data was determined with the Shapiro-Wilk test. If data were normally distributed, a Students t-test was performed to evaluate differences between two groups, and a one-way ANOVA was used to test differences between more than two groups. If normality assumption was not obtained, the Mann-Whitney U test and Kruskal-Wallis test were performed. If the statistical test revealed significant differences between the study groups, post hoc analyses were followed to compare transfusion groups to control group and/or the group receiving standard male RBCs. A p-value of less than 0.05 was considered significant. Unless stated otherwise, reported p-values in the following results section are related to pairwise comparisons of the study groups with the control group and/or the group receiving standard male RBCs. The text presented data in means and standard deviations (SD) and graphs were presented in box plots representing medians and interquartile ranges (IQR). Statistical analyses were performed using SPSS version 26.00 software, and graphical representation was generated using GraphPad Prism version 8.3.0.

## Results

Due to technical issues, two animals were droped out of the group receiving male standard RBCs, yielding n = 8 in this group. All other groups underwent transfusion uneventfully.

### Bloodletting leads to enrichment of reticulocytes in donor blood

**Fig 2:**
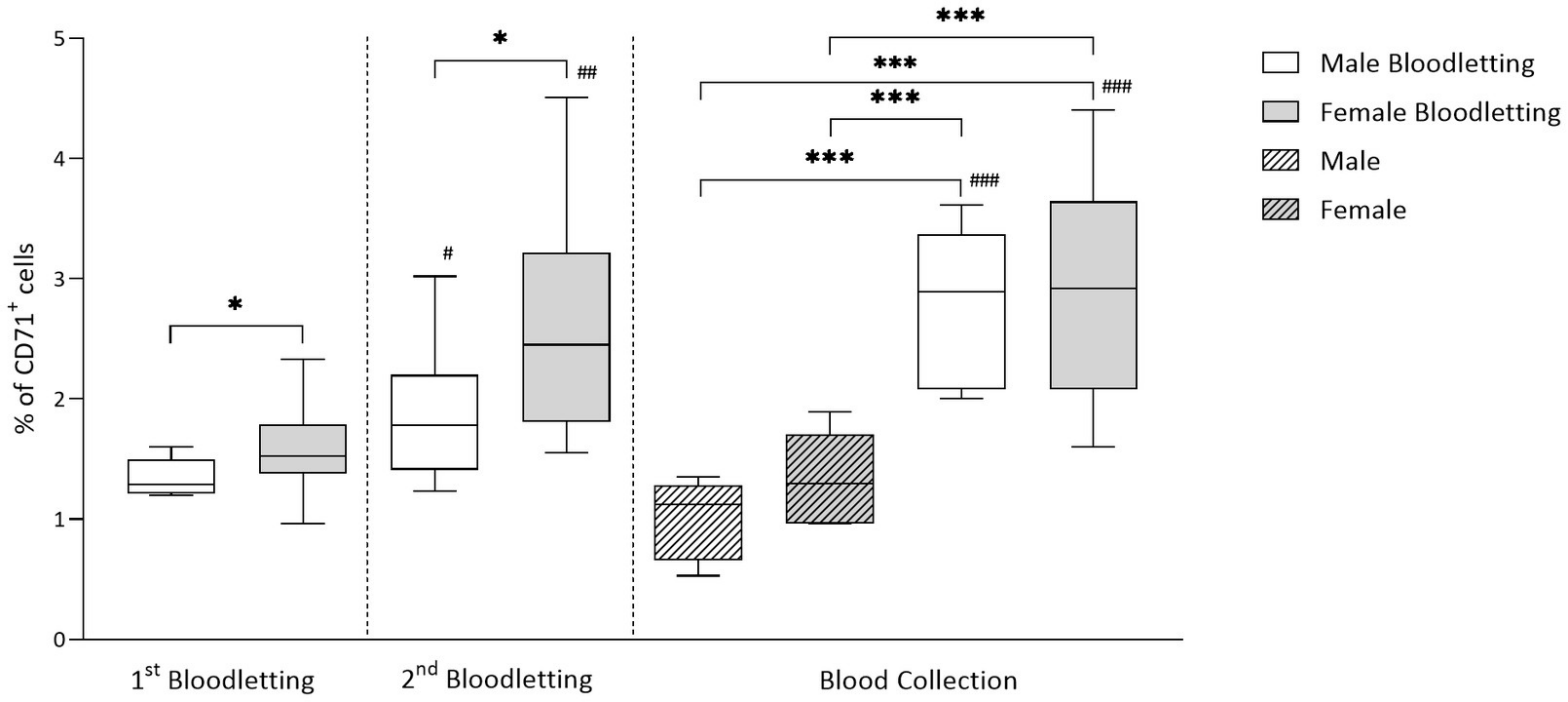
Reticulocyte (CD71^+^ cells) quantification in samples at bloodletting (BL) and in samples of the blood product from male, female, male bloodletting, and female bloodletting donor groups. * denotes a significant difference between groups within the same time point (*P < 0.05, **P < 0.01, ***P < 0.001). ^#^ denotes a significant difference within the same group of bloodletting at the three time points (^#^P < 0.05, ^##^P < 0.01, ^###^P < 0.001).

Characteristics of donor groups at the time of collecting blood products are summarized in S1 Table. The MCV and RDW of RBCs was significantly higher in bloodletting groups compared to non-bloodletting groups for both male and female donors (*p-value* < 0.001). MCHC was significantly higher in non-bloodletting donor groups compared to bloodletting donors (*p-value* < 0.05). Bloodletting led to differences in blood gas parameters in the female group, showing lower pO_2_ and higher lactate and potassium concentrations compared to non-bloodletting groups (*p-value* < 0.001). Fig 2 shows the percentage of reticulocytes in donor samples. Bloodletting led to the enrichment of CD71^+^ cells from 1.3 ± 0.2% to 2.8 ± 0.6% in male donors and from 1.6 ± 0.3% to 2.9 ± 0.9% in female donors (*p-value* < 0.001). Percoll fractionation further enhanced the young RBC fraction, from male donors and from female donors. These products are referred to a ‘young RBCs’. Collected blood from the same group was pooled together into one blood product. All blood products contained similar numbers and characteristics of RBCs (S2 Table).

### Effects of transfusing female and male red blood cells on indices and blood gas analyses

**Fig 3:**
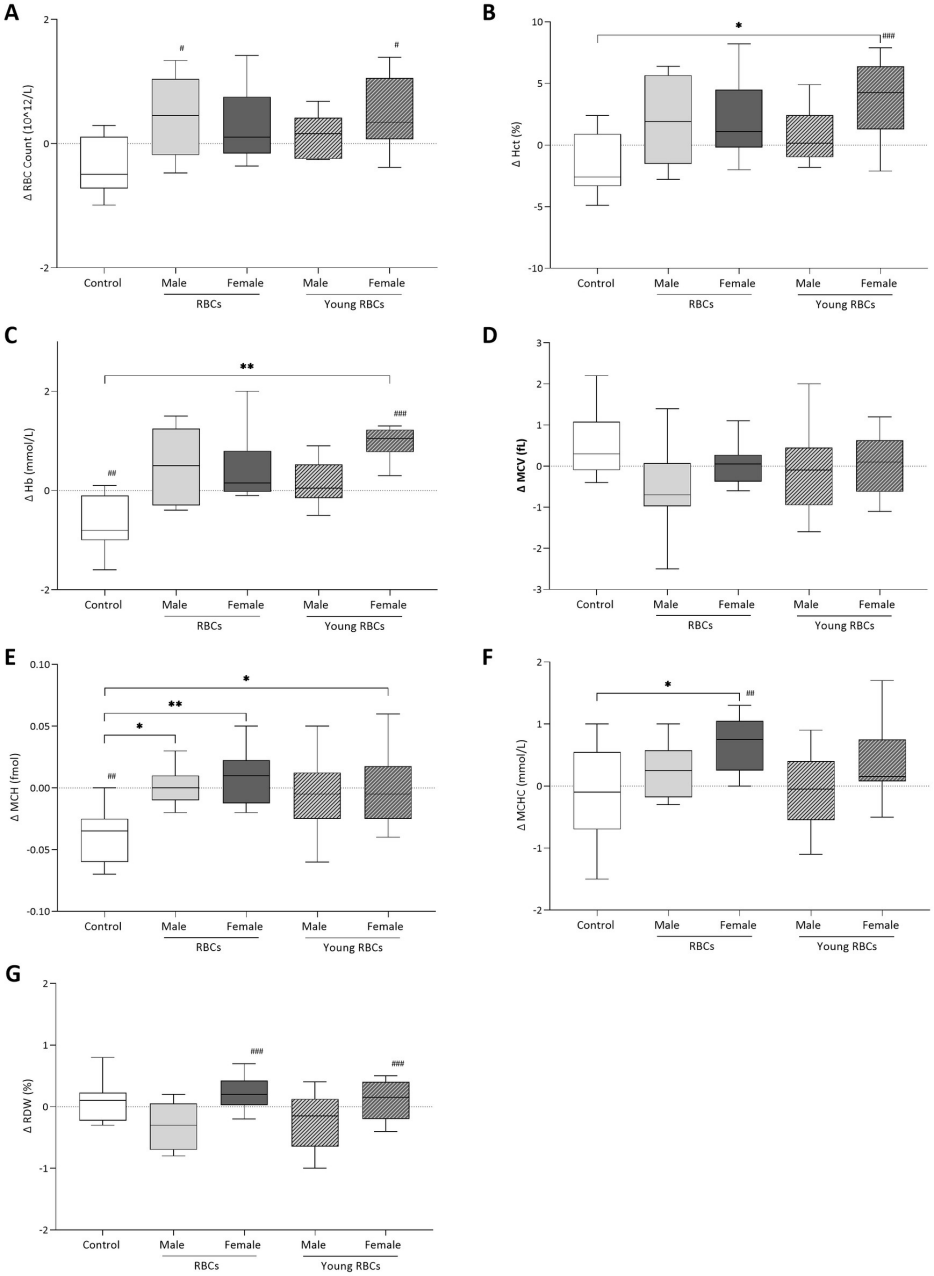
Post-transfusion differences between recipient groups in RBC indices of (A) RBC count, (B) hematocrit (Hct), (C) hemoglobin (Hb) level, (D) mean corpuscular volume (MCV), (E) mean corpuscular hemoglobin (MCH), (F) mean corpuscular hemoglobin concentration (MCHC), and (G) red blood cell distribution width (RDW). Δ is the difference between the pre-transfusion testing and the post-transfusion sample (15 minutes after transfusion) and difference between the pre-transfusion testing and the 24 hours after transfusion sample. Zero line represents no change from pre-transfusion testing. BL stands for bloodletting donors. * denotes a significant difference from control group (*P < 0.05, **P < 0.01, ***P < 0.001). ‡ denotes a significant difference from the group receiving male standard RBCs (‡P < 0.05, ‡‡P < 0.01, ‡‡‡P < 0.001). ^#^ denotes a significant difference from pre-transfusion testing of the same group (^#^P < 0.05, ^##^P < 0.01, ^###^P < 0.001).

**Fig 4:**
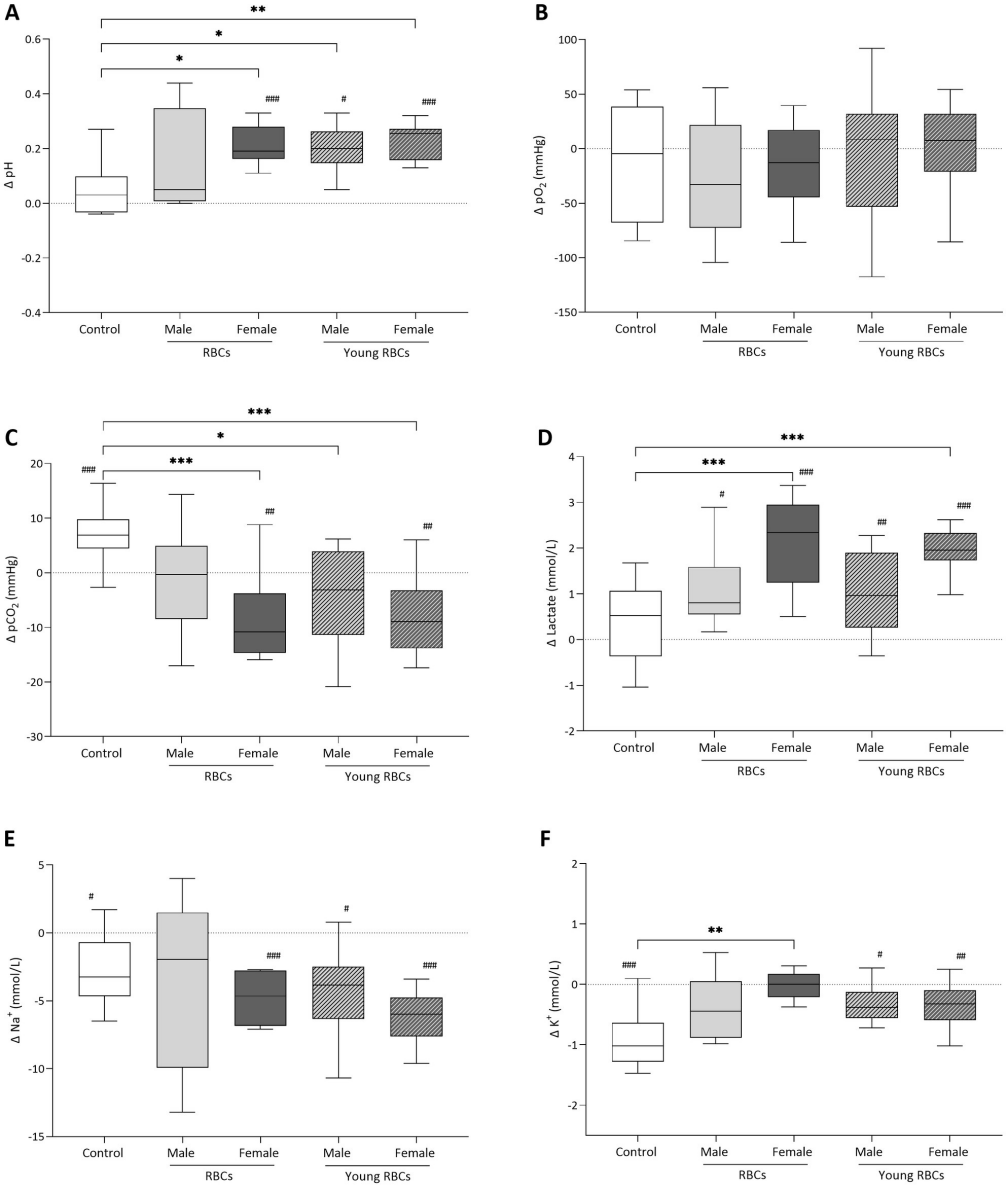
Post-transfusion differences between recipient groups in blood gas parameters of (A) pH, (B) partial pressure of oxygen (pO_2_), (C) partial pressure of carbon dioxide (pCO_2_), (D) lactate concentration, (E) sodium concentration (Na^+^), and (F) potassium concentration (K^+^). Δ is the difference between the pre-transfusion testing and the post-transfusion sample (15 minutes after transfusion) and difference between the pre-transfusion testing and the 24 hours after transfusion sample. Zero line represents no change from pre-transfusion testing. BL stands for bloodletting donors. * denotes a significant difference from control group (*P < 0.05, **P < 0.01, ***P < 0.001). ^#^ denotes a significant difference from pre-transfusion testing of the same group (^#^P < 0.05, ^##^P < 0.01, ^###^P < 0.001).

Baseline characteristics prior to receiving transfusion showed no differences between groups (Table 1). The majority of differences between groups appeared directly after blood transfusion (Figs 3 and 4). Hb concentration and MCH were significantly lower in the post-transfusion of the control group compared to the baseline pre-transfusion sample, probably due to hemodilution following normal saline infusion in this group. No differences were detected between the group receiving standard male RBCs and other transfusion groups except for RDW which was higher in recipients of female standard RBCs compared to the group receiving male standard RBCs (*p-value* < 0.05).

**Table 1:** Baseline characteristics of recipient groups.

	Control (n = 10)	Receiving standard RBCs from		Receiving young RBCs from		*p-value*
		Male (n = 8)	Female (n = 10)	Male BL (n = 10)	Female BL (n = 10)	
**Demographics**						
Age (weeks)	13.2 ± 0.1	13.1 ± 0.2	13.1 ± 0.2	13.2 ± 0.1	13.1 ± 0.2	0.59
Weight (g)	383.8 ± 19.5	378.9 ± 13.1	368.0 ± 17.5	382.8 ± 13.1	372.5 ± 17.6	0.17
**RBC indices**						
RBC count (10^12^/L)	6.3 ± 0.3	6.2 ± 0.8	6.4 ± 0.5	6.2 ± 0.7	6.5 ± 0.4	0.81
Hct (%)	33.9 ± 1.0	34.0 ± 0.8	34.8 ± 2.7	33.8 ± 3.8	34.0 ± 2.0	0.95
Hb (mmol/L)	7.6 ± 0.2	7.4 ± 1.0	7.7 ± 0.6	7.2 ± 0.7	7.2 ± 0.4	0.31
MCV (fL)	53.7 ± 1.0	54.5 ± 1.4	54.4 ± 0.9	54.6 ± 0.8	54.8 ± 1.6	0.27
MCH (fmol)	1.2 ± 0.02	1.2 ± 0.03	1.2 ± 0.02	1.2 ± 0.03	1.2 ± 0.03	0.06
MCHC (mmol/L)	21.6 ± 0.5	21.6 ± 0.5	21.6 ± 0.5	21.4 ± 0.5	21.8 ± 0.5	0.82
RDW (%)	11.7 ± 0.4	12.3 ± 1.3	11.5 ± 0.2	11.8 ± 0.3	12.1 ± 0.8	0.13
**Blood gas**						
pH	7.0 ± 0.1	7.0 ± 0.3	7.0 ± 0.04	6.9 ± 0.1	7.0 ± 0.03	0.12
pO_2_ (mmHg)	157.6 ± 8.8	176.1 ± 27.5	172.5 ± 26.0	168.4 ± 27.7	172.0 ± 14.1	0.42
pCO_2_ (mmHg)	37.3 ± 3.0	42.5 ± 9.0	39.9 ± 7.8	43.1 ± 6.7	39.6 ± 4.1	0.28
Lactate (mmol/L)	2.9 ± 0.7	2.2 ± 0.5	2.7 ± 0.5	2.4 ± 0.3	2.5 ± 0.5	0.05
Na^+^ (mmol/L)	151.0 ± 1.4	148.9 ± 6.9	151.1 ± 2.5	152.3 ± 2.9	150.6 ± 1.0	0.34
K^+^ (mmol/L)	4.5 ± 0.4	4.4 ± 1.4	4.1 ± 0.2	4.2 ± 0.3	4.3 ± 0.3	0.60
**Proportion of transfused RBCs in circulation** *	—	7.0 ± 1.1	6.7 ± 1.4	6.6 ± 1.0	6.8 ± 1.4	0.92

BL, bloodletting; Hct, hematocrit; Hb, hemoglobin level; MCV, mean corpuscular volume; MCH, mean corpuscular hemoglobin; MCHC, mean corpuscular hemoglobin concentration; RDW, red blood cell distribution width; pO_2_, partial pressure of oxygen; pCO_2_, partial pressure of carbon dioxide; Na^+^, sodium concentration; K+, potassium concentration. Data are presented in mean ± SD.

*calculated from the percentage of biotinylated RBC in the circulation at the 15-min post-transfusion time point

### Receipt of female blood enriched for young RBCs may be associated with lower PTR compared to receipt of male blood

**Fig 5:**
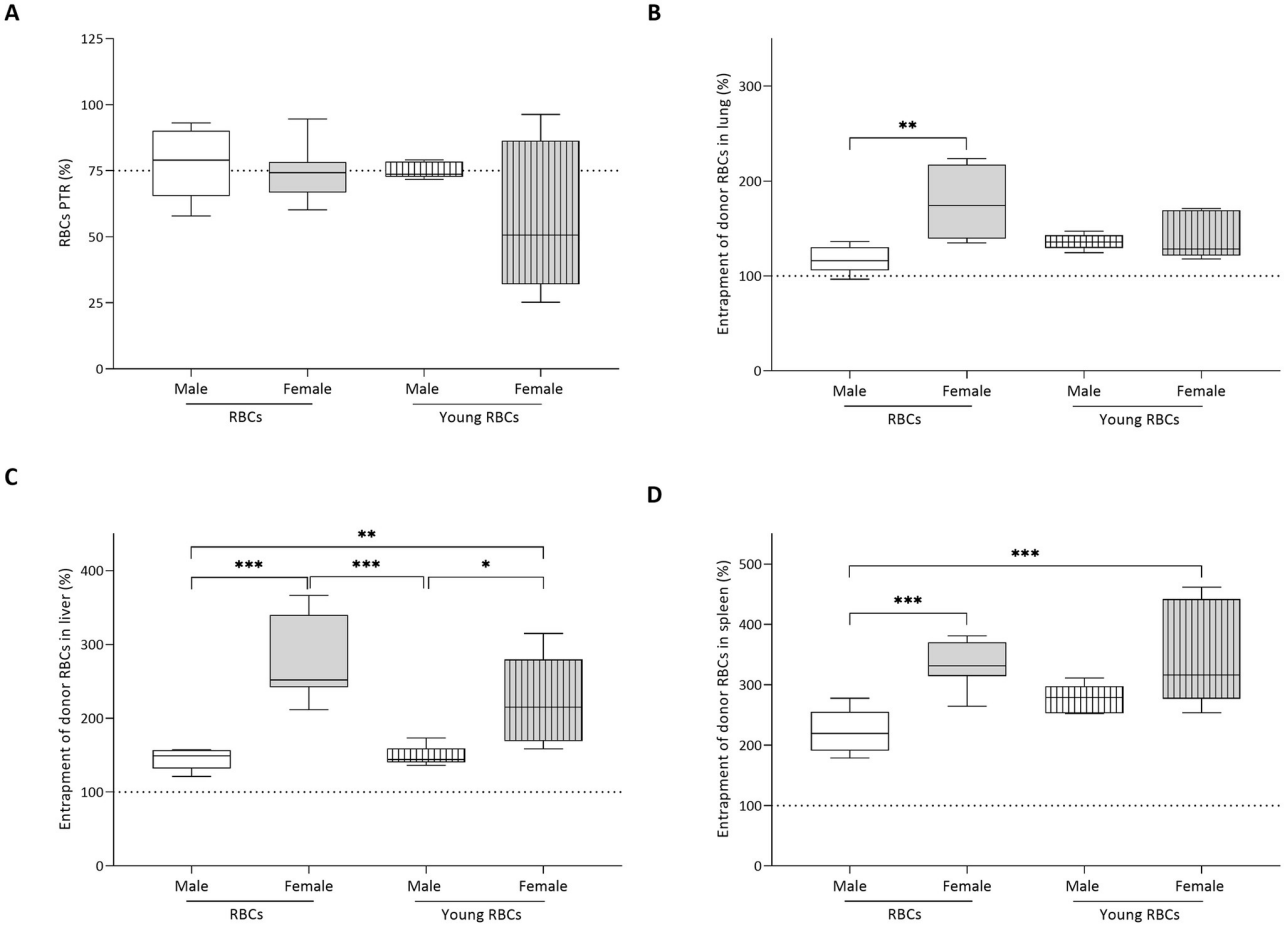
(A) Post transfusion recovery (PTR). Box plot represents the median and interquartile range (IQR) of the percentages of donor RBCs in recipients’ blood at 24 hours after transfusion. The line represents the lower limit of the regulations for transfusing human RBCs (75% PTR). Entrapment of donor RBCs in recipients’ (B) lung, (C) liver, and (D) spleen compared to the circulation at 24 hours after transfusion. The 100% percentage line represents the donor RBCs in the circulation. * denotes a significant difference from the group receiving male standard RBCs (*P < 0.05, **P < 0.01, ***P < 0.001).

Only recipients of female young RBCs did not reach a PTR threshold of 75% (Fig 5) and mean PTR tended to be lower (62.5 ± 29.6%) than the group receiving male standard RBCs (77.5 ± 12.8%), although not reaching statistical significance (*p-value* = 0.07).

### Receipt of female blood is associated with increased entrapment of donor RBCs in organs irrespective of the age of RBCs

Following transfusion, a small amount of donor RBCs was found in organs in all donor groups when compared to the circulation (Fig 5). Compared to recipients of standard RBCs from male blood (116.7 ± 13.9%), recipients of standard RBCs from female donors showed increased amounts of donor RBCs in lung (176.9 ± 32.5%) (*p-value* < 0.01). In liver, recipients of standard RBCs from female donors (277.9 ± 49.7%) as well as young RBCs from female donors (225.5 ± 54.1%) were associated with increased entrapment of transfused RBCs compared to recipients of male standard RBCs (151.0 ± 25.5%) (*p-value* < 0.001 and *p-value* < 0.01, respectively). Similarly, recipients of standard RBCs from female donors (334.8 ± 33.5%) as well as young RBCs from female donors (345.5 ± 81.7%) were associated with increased entapment of donor RBCs in spleen compared to recipients of standard RBCs from male blood (222.9 ± 34.9%) (*p-value* < 0.001).

### Receipt of female blood is associated with increased hemolysis and increased plasma concentration of soluble ICAM-1, irrespective of the age of RBCs

**Fig 6:**
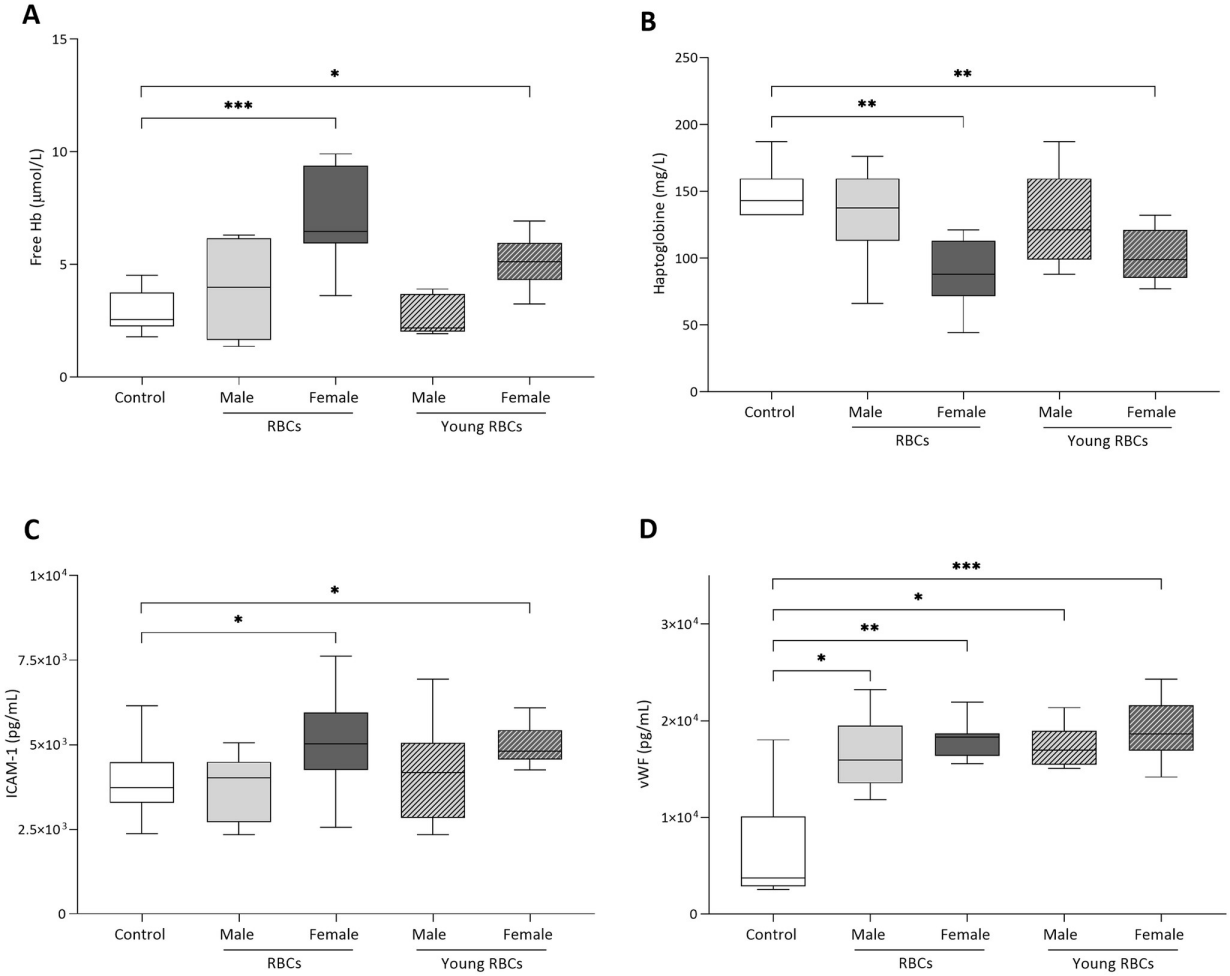
Concentrations of (A) free hemoglobin (Hb), (B) haptoglobin, (C) soluble ICAM-1 (sICAM-1), and (D) von Willebrand Factor antigen (vWF) in the plasma at 24 hours after transfusion. BL stands for bloodletting donors. * denotes a significant difference from control group (*P < 0.05, **P < 0.01, ***P < 0.001). ‡ denotes a significant difference from the group receiving male standard RBCs (‡P < 0.05, ‡‡P < 0.01, ‡‡‡P < 0.001).

Free Hb concentration was significantly higher in the group receiving female standard RBCs (7.1 ± 2.0 μmol/L) and in the group receiving young female RBCs (5.3 ± 1.7 μmol/L) compared to the control group (2.9 ± 0.9 μmol/L) (Fig 6, *p-value* < 0.001 and *p-value* < 0.05, respectively). Haptoglobin was significantly lower in the group receiving female standard RBCs (93.5 ± 36.8 mg/L) and in the group receiving female young RBCs (102.3 ± 18.7 mg/L) compared to the control group (143.0 ± 30.2 mg/L) (*p-value* < 0.01 and *p-value* < 0.05, respectively). Concentration of soluble ICAM-1 was significantly higher in recipients of female standard RBCs (5.0 ± 1.4 ng/mL) and female young RBCs (5.4 ± 1.7 ng/mL) compared to the control group (4.0 ± 1.1 ng/mL) and male standard RBCs (3.7 ± 0.9 ng/mL) (*p-value* < 0.05). The level of vWF was higher in all groups compared to the control group (*p-value* < 0.05), but did not differ between groups receiving a blood transfusion.

### Receipt of female blood is associated with higher splenic iron content compared to receipt of male blood

The histological assessment showed no pathological abnormalities in the lung, liver, and spleen after 24 hours of transfusion (S1 Fig). There was a trend towards increased lung injury in the group receiving male standard RBCs compared to the control group (*p-value = 0*.*06*). However, these pathological injuries are considered very mild. In addition, the splenic iron content of the group receiving standard female RBCs (18.8 ± 1.7%) was higher compared to the group receiving standard male RBCs (14.8 ± 5.2%) (*p-value* < 0.05).

## Discussion

The major findings of this animal study are that transfusion of female blood is associated with increased entrapment of transfused RBCs in organs, accompanied by increased endothelial activation and hemolysis. These findings are irrespective of the amount of young cells in the product. In addition, only transfusion of young RBCs from females reduced the PTR threshold below 75%.

To ensure oxygen delivery to organs, RBCs must circulate freely without obstructing or inadvertently interacting with surrounding cells. We offer differential explanations for our finding that transfusion of female RBCs is associated with higher organ entrapment of donor cells as well as increased levels of soluble ICAM-1 and increased hemolysis. Possibly, donor cells adhere to the endothelial cell wall, get entrapped in the microvasculature of organs, followed by hemolysis and release of free Hb into the circulation. In accordance with this hypothesis, previous studies have demonstrated that RBCs and sickle cells can interact with endothelial cells via ICAM-1, associated with hemolysis [27, 28]. An alternative hypothesis is that female RBCs [17] have a different phenotype than male RBCs, rendering them susceptible to phagocytosis following transfusion. As the RBC count directly following transfusion of female blood in our study was higher than the baseline, we can imply that hemolysis did not occur directly following transfusion but rather within the 24 h post-transfusion period. This would result in the overloading of the reticuloendothelial system (RES) and releasing of free hemoglobin into the circulation. In line, we found an increased iron content in the spleen following transfusion of female blood. A third explanation is that female RBCs may become susceptible to hemolysis following transfusion into male recipients due to a soluble factor, such as testosterone. Possibly in line with this, a previous study showed that testosterone increased the risk of hemolysis during storage and after transfusion to male mice [29]. Also, some observational studies show an association between transfusion of female blood with increased mortality of male recipients, which was most prominent in men younger then 50 years of age [7, 30, 31].

In contrast to our hypothesis, endothelial activation and organ entrapment of donor cells did not depend on the number of young RBCs in the donor blood. This is not in line with a previous *in vitro* study that suggested that young RBCs are involved in endothelial adherence and activation [28].

The current study reported a trend towards lower PTR following blood from female donors with a high fraction of young RBCs as compared to other groups. This finding is contradicting previous studies that showed that transfusion of young RBCs is associated with a higher survival compared to standard transfusion [32–36]. It is unlikely that the bloodletting is the reason for a lower RBC count, as the RBC count directly after transfusion of young RBCs from female was higher compared to the baseline testing. Therefore, the low PTR may be due to higher clearance from the circulation, which is in agreement with the trapping of the RBCs within the organs. Another explanation for the lower PTR in the group receiving female young RBCs may be related to the high level lactate and K^+^ in the female bloodletting group at the time of blood collection, which may reflect an impact from the bloodletting procedure. Of note, the present study in rats showed overall a low PTR. A possible explanation might be related to using normovolemic healthy recipients. Approximately 10% of "unneeded" blood was infused into the circulation, which could be removed partially from the circulation. We chose a normovolemic rat model in an effort to delineate results solely attributable to the transfusion products but not to any other underlaying pathology within the recipients. This hampers extrapolation to a clinically relevant setting.

An obvious limitation of this study is extrapolation of findigns of blood letting in mice to menstrual losses in humans. In addition, the use of a non-anemic model limits the generalisability of our findings to an anemic setting. Of note, all recipients were males, as we previously noted an adverse outcome of male patients receiving female blood, which was absent in female recipients [7]. Hereby, whether findings in this study are due to sex-mismatched transfusion or to the receipt of female blood can not be determined. Furthermore, although the method to enrich young cells was successful, the percentage of CD71^+^ cells was still low. Another limitation in the methodology of this study is related to assuming that all transfused RBCs are structurally intact. It could be that the extra steps of centrifugation for bloodletting donors causes more fragmented cells. Moreover, this study only evaluated RBC clearance and organ entrapment at one time point (24 hours), and we do not know if those outcomes differ at earlier time points. Lastly, the direct mechanisms of RBC entrapment in organs, endothelial activation, and hemolysis, and the order of events, was not investigated in this study. Future studies with more focus on the mechanisms are therefore recommended.

This work contributes to our understanding of the impact of receiving blood from female donors on transfusion outcomes [7–9]. In summary, transfusing both young and standard RBCs from female donors, but not from male donors, is associated with trapping of donor RBCs in organs of male recipients, accompanied by endothelial activation and hemolysis. Our results confirm that donor sex affects transfusion outcomes.

## Supporting information

S1 Table Characteristics of pooled blood product at the time of transfusion. (DOCX)

S2 Table Baseline characteristics of recipient groups before receiving the blood product. (DOCX)

S1 Fig Histology scores for (A) lung, (B) liver, and (C) spleen. (D) Splenic iron accumulation after 24 hours of transfusion. BL stands for bloodletting donors. * denotes a Significant difference.
(TIF)

S1 Dataset (XLSX)
