# Supplementary material for: Transfusion of female blood in a rat model is associated with red blood cells entrapment in organs
Source: PLoS One. 2023 Nov 22;18(11):e0288308. doi: 10.1371/journal.pone.0288308 (PMC10664878; doi:10.1371/journal.pone.0288308)
Supplement: S1 Table — (DOCX) [file pone.0288308.s001.docx]

**S1 Table. Characteristics of pooled blood product at the time of transfusion**

|  | Pooled standard RBCs from | |  | Pooled young RBCs from | |
| --- | --- | --- | --- | --- | --- |
|  | Male  (n = 5) | Female  (n = 8) |  | Male BL  (n = 11) | Female BL  (n = 14) |
| **RBC indices** |  |  |  |  |  |
| RBC count (10^12^/L) | 6.8 | 6.7 |  | 6.4 | 6.3 |
| Hct (%) | 37.7 | 38.7 |  | 37.6 | 38.1 |
| Hb (mmol/L) | 8.4 | 8.8 |  | 7.8 | 8.4 |
| MCV (fL) | 55.5 | 57.5 |  | 56.3 | 59.4 |
| MCH (fmol) | 1.2 | 1.3 |  | 1.2 | 1.3 |
| MCHC (mmol/L) | 21.1 | 22.9 |  | 21.7 | 22.6 |
| RDW (%) | 11.8 | 11.2 |  | 15.3 | 13.8 |
| **Blood gas** |  |  |  |  |  |
| pH | 6.7 | 6.6 |  | 6.7 | 6.7 |
| pO_2_ (mmHg) | 261.7 | 261.5 |  | 211.4 | 232.0 |
| pCO_2_ (mmHg) | 6.9 | 8.9 |  | 8.6 | 7.6 |
| Lactate (mmol/L) | 1.2 | 1.7 |  | 1.4 | 2.3 |
| Na^+^ (mmol/L) | 143.6 | 144.6 |  | 145.7 | 144.9 |
| K^+^ (mmol/L) | 4.6 | 4.6 |  | 4.1 | 5.2 |
| **Reticulocyte quantification** |  |  |  |  |  |
| CD71+ cells | 1.1 | 1.4 |  | 3.8 | 4.3 |
