# Supplementary material for: Transfusion of female blood in a rat model is associated with red blood cells entrapment in organs
Source: PLoS One. 2023 Nov 22;18(11):e0288308. doi: 10.1371/journal.pone.0288308 (PMC10664878; doi:10.1371/journal.pone.0288308)
Supplement: S2 Table — (DOCX) [file pone.0288308.s002.docx]

**S2 Table. Baseline characteristics of recipient groups before receiving the blood product**

|  | Control | Receiving RBCs from | |  | | Receiving young RBCs from | | *p-value* |
| --- | --- | --- | --- | --- | --- | --- | --- | --- |
|  |  | Male | Female | |  | Male BL | Female BL |  |
| **Demographics** |  |  |  | |  |  |  |  |
| Age (weeks) | 13.2 ± 0.1 | 13.1 ± 0.2 | 13.1 ± 0.2 | |  | 13.2 ± 0.1 | 13.1 ± 0.2 | 0.59 |
| Weight (g) | 383.8 ± 19.5 | 378.9 ± 13.1 | 368.0 ± 17.5 | |  | 382.8 ± 13.1 | 372.5 ± 17.6 | 0.17 |
| **RBC indices** |  |  |  | |  |  |  |  |
| RBC count (10^12^/L) | 6.3 ± 0.3 | 6.2 ± 0.8 | 6.4 ± 0.5 | |  | 6.2 ± 0.7 | 6.5 ± 0.4 | 0.81 |
| Hct (%) | 33.9 ± 1.0 | 34.0 ± 0.8 | 34.8 ± 2.7 | |  | 33.8 ± 3.8 | 34.0 ± 2.0 | 0.95 |
| Hb (mmol/L) | 7.6 ± 0.2 | 7.4 ± 1.0 | 7.7 ± 0.6 | |  | 7.2 ± 0.7 | 7.2 ± 0.4 | 0.31 |
| MCV (fL) | 53.7 ± 1.0 | 54.5 ± 1.4 | 54.4 ± 0.9 | |  | 54.6 ± 0.8 | 54.8 ± 1.6 | 0.27 |
| MCH (fmol) | 1.2 ± 0.02 | 1.2 ± 0.03 | 1.2 ± 0.02 | |  | 1.2 ± 0.03 | 1.2 ± 0.03 | 0.06 |
| MCHC (mmol/L) | 21.6 ± 0.5 | 21.6 ± 0.5 | 21.6 ± 0.5 | |  | 21.4 ± 0.5 | 21.8 ± 0.5 | 0.82 |
| RDW (%) | 11.7 ± 0.4 | 12.3 ± 1.3 | 11.5 ± 0.2 | |  | 11.8 ± 0.3 | 12.1 ± 0.8 | 0.13 |
| **Blood gas** |  |  |  | |  |  |  |  |
| pH | 7.0 ± 0.1 | 7.0 ± 0.3 | 7.0 ± 0.04 | |  | 6.9 ± 0.1 | 7.0 ± 0.03 | 0.12 |
| pO_2_ (mmHg) | 157.6 ± 8.8 | 176.1 ± 27.5 | 172.5 ± 26.0 | |  | 168.4 ± 27.7 | 172.0 ± 14.1 | 0.42 |
| pCO_2_ (mmHg) | 37.3 ± 3.0 | 42.5 ± 9.0 | 39.9 ± 7.8 | |  | 43.1 ± 6.7 | 39.6 ± 4.1 | 0.28 |
| Lactate (mmol/L) | 2.9 ± 0.7 | 2.2 ± 0.5 | 2.7 ± 0.5 | |  | 2.4 ± 0.3 | 2.5 ± 0.5 | 0.05 |
| Na^+^ (mmol/L) | 151.0 ± 1.4 | 148.9 ± 6.9 | 151.1 ± 2.5 | |  | 152.3 ± 2.9 | 150.6 ± 1.0 | 0.34 |
| K^+^ (mmol/L) | 4.5 ± 0.4 | 4.4 ± 1.4 | 4.1 ± 0.2 | |  | 4.2 ± 0.3 | 4.3 ± 0.3 | 0.60 |

BL, bloodletting; Hct, hematocrit; Hb, hemoglobin level; MCV, mean corpuscular volume; MCH, mean corpuscular hemoglobin; MCHC, mean corpuscular hemoglobin concentration; RDW, red blood cell distribution width; pO_2_, partial pressure of oxygen; pCO_2_, partial pressure of carbon dioxide; Na^+^, sodium concentration; K+, potassium concentration. Data are presented in mean ± SD.
