# Supplementary figures and images for: Transfusion of female blood in a rat model is associated with red blood cells entrapment in organs
Source: PLoS One. 2023 Nov 22;18(11):e0288308. doi: 10.1371/journal.pone.0288308 (PMC10664878; doi:10.1371/journal.pone.0288308)

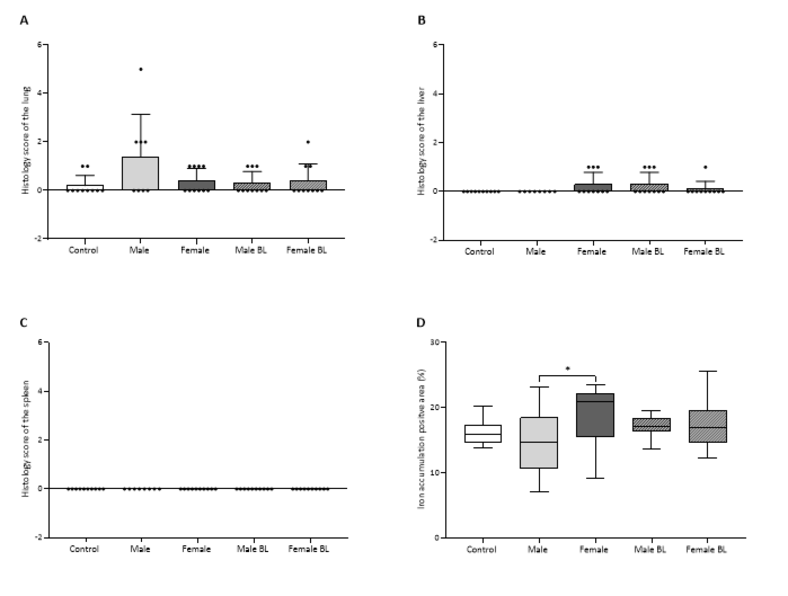

Supplement: S1 Fig — Histology scores for (A) lung, (B) liver, and (C) spleen. (D) Splenic iron accumulation after 24 hours of transfusion. BL stands for bloodletting donors. * denotes a Significant difference. (TIF) [file pone.0288308.s003.tif]
